# Robot-Assisted Orbital Fat Decompression Surgery: First in Human

**DOI:** 10.1167/tvst.11.5.8

**Published:** 2022-05-10

**Authors:** Yi Wang, Jing Sun, Xingtong Liu, Yinwei Li, Xianqun Fan, Huifang Zhou

**Affiliations:** 1 Department of Ophthalmology, Shanghai Ninth People's Hospital, Shanghai JiaoTong University School of Medicine, Shanghai, China

**Keywords:** robot-assisted orbital fat decompression surgery, da Vinci surgical system, exophthalmos, GO-QoL

## Abstract

**Purpose:**

To explore the safety and feasibility of robot-assisted orbital fat decompression surgery.

**Methods:**

Ten prospectively enrolled patients (18 eyes) with Graves’ ophthalmopathy underwent robot-assisted orbital fat decompression surgery with the da Vinci Xi surgical system. Intraoperative blood loss, operative time, and complications were recorded. For every patient, the exophthalmos of the operated eyes and Graves’ orbitopathy quality of life (GO-QoL) were measured both preoperatively and 3 months postoperatively to assess the surgical effect.

**Results:**

All surgical procedures were successfully performed. The mean duration to complete the whole procedure was 124.3 ± 33.2 minutes (range, 60–188). The mean intraoperative blood loss was 17.8 ± 6.2 mL (range, 7.5–28). There were neither complications nor unexpected events in terms of either orbital decompression surgery or robot-assisted procedures. The mean exophthalmos was 20.2 ± 1.8 mm before surgery and 17.9 ± 1.4 mm postoperatively (*P* < 0.0001). The preoperative and postoperative GO-QoL on the visual function arm was 84.38 ± 20.04 and 93.75 ± 9.32, respectively. The preoperative and postoperative GO-QoL on the appearance arm was 42.50 ± 14.97 and 64.38 ± 21.46, respectively (*P* = 0.027).

**Conclusions:**

The da Vinci Xi surgical system provided the stability, dexterity, and good visualization necessary for orbital fat decompression surgery, indicating the safety and feasibility of robot-assisted orbital fat decompression surgery.

**Translational Relevance:**

Based on a literature search using EMBASE and MEDLINE databases, we believe that this study reports the first in-human results of the safety and effectiveness of da Vinci robot-assisted orbital fat decompression surgery.

## Introduction

Graves’ orbitopathy (GO) is the most prevalent orbital disease worldwide.^1^^,^^2^ It is the most common extra-thyroid symptom of Graves’ disease^3^ and is characterized by autoimmune inflammation of orbital tissues leading to retro-ocular tissue hyperplasia and eyeball proptosis. For patients with GO who enter the resting stage of the disease with proptosis affecting appearance and visual function, orbital decompression surgery is the first-option treatment according to GO treatment guidelines European Group on Graves’ Orbitopathy (EUGOGO) 2021.^4^^,^^5^ Orbital fat decompression surgery is performed on patients with GO who have exophthalmos dominantly attributed to hyperplasia of orbital fat. With the evolution of the surgical technique, the indications for this surgery have expanded. It has been shown to be of functional and cosmetic benefit to relative proptosis of nonthyroid origin^6^^–^^9^ and is also performed on normal people who are bothered by the prominence of their eyes and desire aesthetic improvement.^7^^,^^10^ Thus, higher requirements for surgical precision, minimal invasion, and effect have been proposed.

Orbital fat decompression surgery with a manual procedure has disadvantages, such as a small surgical field, unavoidable physiologic trembling, and surgeon fatigue. There is a risk of damaging important anatomic structures, such as blood vessels, nerves, and extraocular muscles, and causing intraoperative or postoperative complications.

Robot-assisted surgery has the advantages of increased precision, greater magnification, scalability of motion, and tremor filtration.^11^^,^^12^ Robot-assisted eye surgery has the potential to reduce tissue damage and increase surgery precision.^13^^,^^14^ Currently, the most widely used robotic surgical system in clinical practice is the da Vinci Surgical System (Intuitive Surgical, Inc., Sunnyvale, CA), which is approved by the US Food and Drug Administration for human surgery. To address the difficulties of traditional manually performed surgery, we introduced the da Vinci robotic system into orbital fat decompression surgery. This study aimed to investigate the safety and feasibility of robot-assisted orbital fat decompression surgery using the da Vinci Xi Surgical System.

## Materials and Methods

### Robot

**Figure 1.**
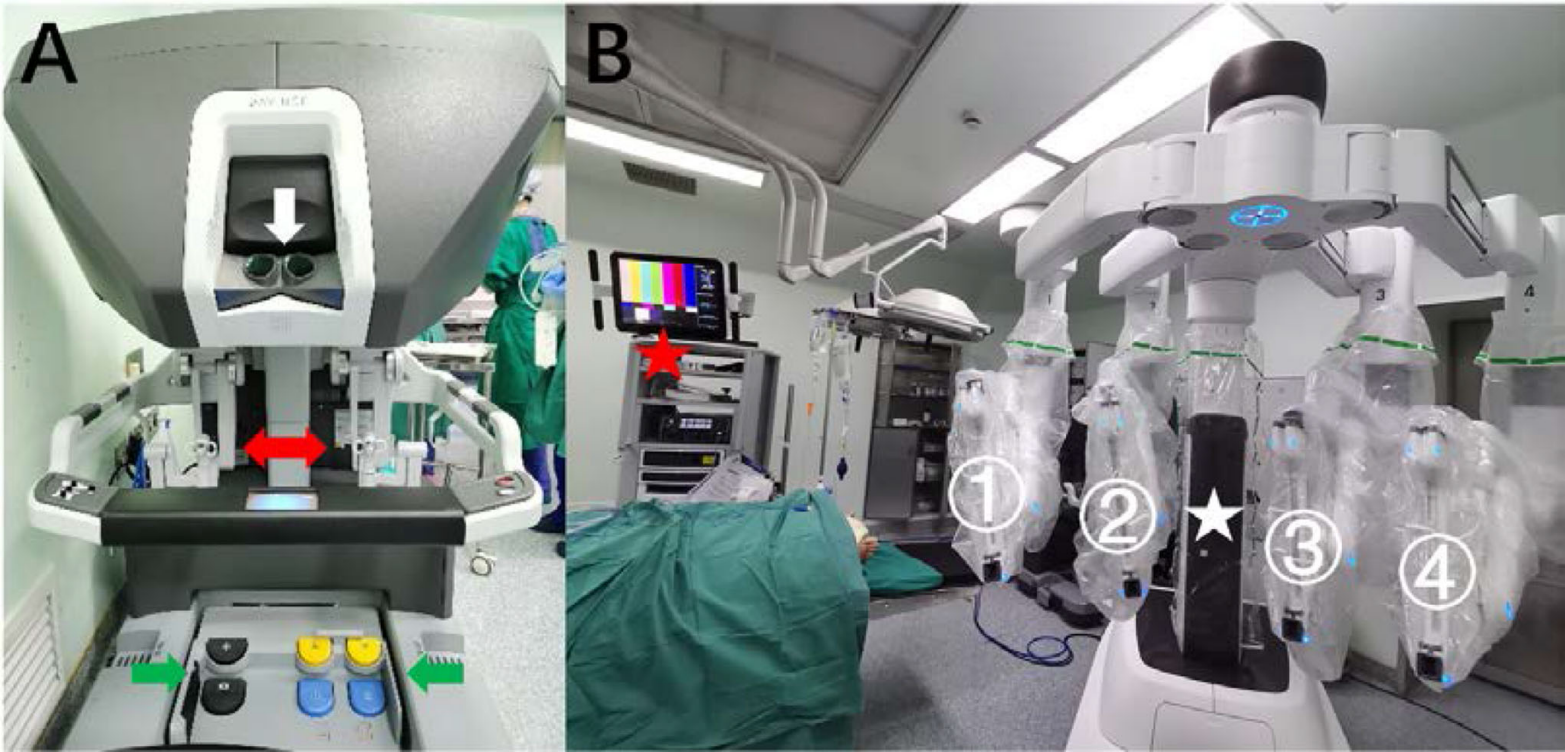
The da Vinci Xi Surgical System. The surgeon's console is equipped with an optical viewing system (**A**, *white arrow*), two telemanipulation handles (**A**, *red arrow*), and five pedals (**A**, *green arrows*). The mobile instrument cart has four articulated arms, ready for loading surgical tools and the endoscope (**B**, *white star*). The vision cart is connected to the robot system (**B**, *red star*).

We used the da Vinci Xi Surgical System in our study. It consists of three components: a mobile instrument cart with four articulated arms that hold three detachable surgical tools and one endoscope, a vision cart, and a surgeon's console to control the robotic arms remotely^15^ (Fig. 1). The camera of the endoscope provides three-dimensional vision with progressive magnification up to 10 times and can autofocus. The computer processor filters hand tremor, and the surgical movements were scaled to 3:1 for delicate manipulation.

### Patients

The study was approved in advance by the ethics committee of Shanghai Ninth People's Hospital, Shanghai, China (clinical trial registration number: ChiCTR2100043768). Patients with GO who entered the inactive stage (clinical activity score <3) according to the EUGOGO guideline 2021 and required orbital fat decompression surgery due to existing exophthalmos were included. Patients who had any of the following conditions were excluded: (1) severe cardiac, hepatic, renal insufficiency, and other surgical contraindications; (2) diabetes or other autoimmune diseases, uncontrolled hypertension, or mental diseases; (3) insufficient self-judgment ability; and (4) pregnancy.

Ten patients with GO (18 eyes) were prospectively enrolled to participate (see detailed information in the Table). All patients were women, who tend to have less severe conditions than men according to the GO epidemiology.^16^ Patients’ ages ranged from 23 to 47 years with a mean age of 30 years. The patients underwent robot-assisted orbital fat decompression surgery between March and June 2021 and conformed to the Declaration of Helsinki. A statement of consent to publish these results and images has been gathered from all patients. The exophthalmos was measured by using Mimics 16.0 software (Materialize, Shanghai, China) based on the computed tomography (CT) scan of the orbits. A standard GO quality-of-life (GO-QoL) questionnaire (see Supplementary Material S1) was introduced to measure the alterations in visual function and appearance on a 0 to 100 scale, with higher values indicating a better result.^17^ The exophthalmos and GO-QoL on visual function and appearance arm were all compared pre- and postoperatively. The follow-up time was 3 months.

**Table.** Detailed Information of Every Patient Receiving da Vinci Robot-Assisted Orbital Fat Decompression Surgery

Patient No.	Age Range, y	Gender	Operated Eye(s)	Preope-rative CAS	Preope-rative Exophthalmos, mm	Preope-rative GO-QoL (Visual Function)	Preope-rative GO-QoL (Appearance)	Total Operative Time, min	Robot Preparation Time, min	Robotic Operation Time, min	Time for Exposure & Suture	Intraop-erative Blood Loss, mL	Removed Fat Volume, mL	Compli-cations	Postope-rative Exophthalmos, mm	Preoper-ative GO-QoL (Visual Function)	Preoper-ative GO-QoL (Appearance)
1	40s	Female	OS	2	17.7	56.25	31.25	116	26	67	23	25	1.3	None	14.3	87.5	62.5
2	20s	Female	OD	0	22.2	68.75	25	188	9	173	6	12	2.6	None	19.2	100	56.25
			OS	0	22.0								3.5		18.3		
3	30s	Female	OD	0	20.3	100	37.5	160	12	142	6	18	3.3	None	18.5	100	68.75
			OS	0	21.0								3.5		18.8		
4	20s	Female	OD	1	18.1	75	68.75	137	21	105	11	23	3.0	Mild pain (VAS = 3)	17.2	100	31.25
			OS	1	19.9								3.4		18.0		
5	30s	Female	OD	1	19.0	100	43.75	104	10	81	13	13	3.2	None	17.8	100	87.5
			OS	1	18.0								3.1		16.6		
6	30s	Female	OD	0	20.9	93.75	43.75	122	7	102	13	17	4.5	None	17.6	93.75	43.75
			OS	0	19.6								3.7		17.9		
7	20s	Female	OD	0	20.9	100	50	130	11	109	10	28	4.2	None	19.7	100	93.75
			OS	0	21.0								4.6		20.2		
8	20s	Female	OD	0	22.0	100	56.25	112	6	100	6	17	4.7	Mild pain (VAS = 2)	19.3	75	93.75
			OS	0	20.4								4.1		17.7		
9	20s	Female	OD	0	22.4	100	50	109	10	84	15	17	5.1	Mild pain (VAS = 2)	18.0	100	50
			OS	0	21.5								5.2		17.2		
10	30s	Female	OD	0	16.2	50	18.75	65	10	50	5	7.5	2.4	None	15.8	81.25	56.25
Mean ± SD	30.0 ± 6.8	NA	NA	0.3 ± 0.6	20.2 ± 1.8	84.38 ± 20.04	42.50 ± 14.97	124.3 ± 33.2	12.2 ± 6.3	101.3 ± 35.6	10.8 ± 5.6	17.8 ± 6.2	3.6 ± 1.0	NA	17.9 ± 1.4	93.75 ± 9.32	64.38 ± 21.46

CAS, clinical activity score; NA, not applicable; OD, right eye; OS, left eye; VAS, visual analog scale (see Supplementary Material S2), which was recorded on the day after surgery for every patient.

### Surgical Procedure

Before surgery, all patients were assessed for orbital fat volume by CT by an experienced doctor using Mimics 16.0 software. A surgical plan that included the surgical route was made, and the volume of fat tissues to be removed was calculated according to the target exophthalmos regression based on the linear correlation between them, which our team described in previous research.^18^

**Figure 2.**
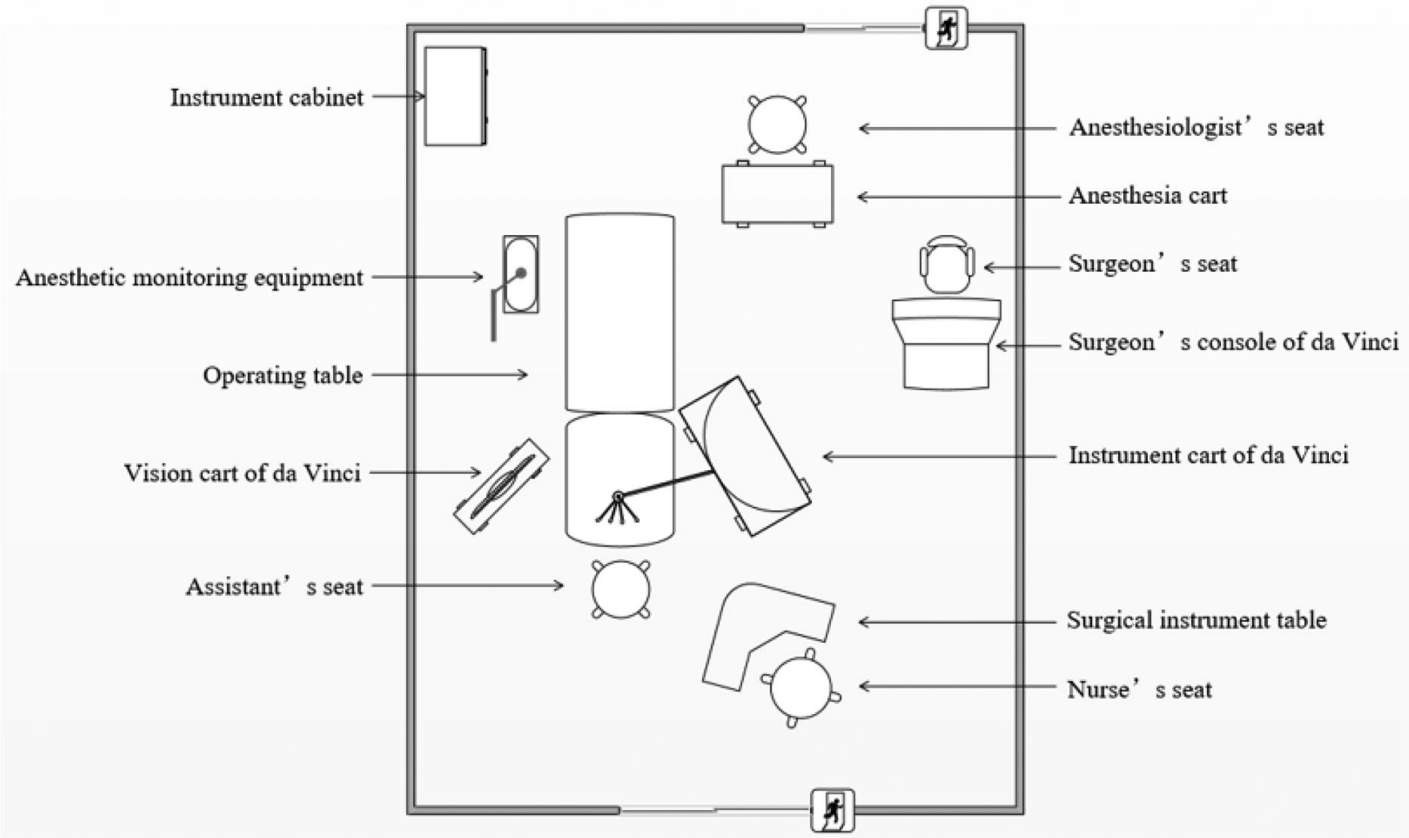
The schematic diagram of the spatial arrangement of the operation room.

**Figure 3.**
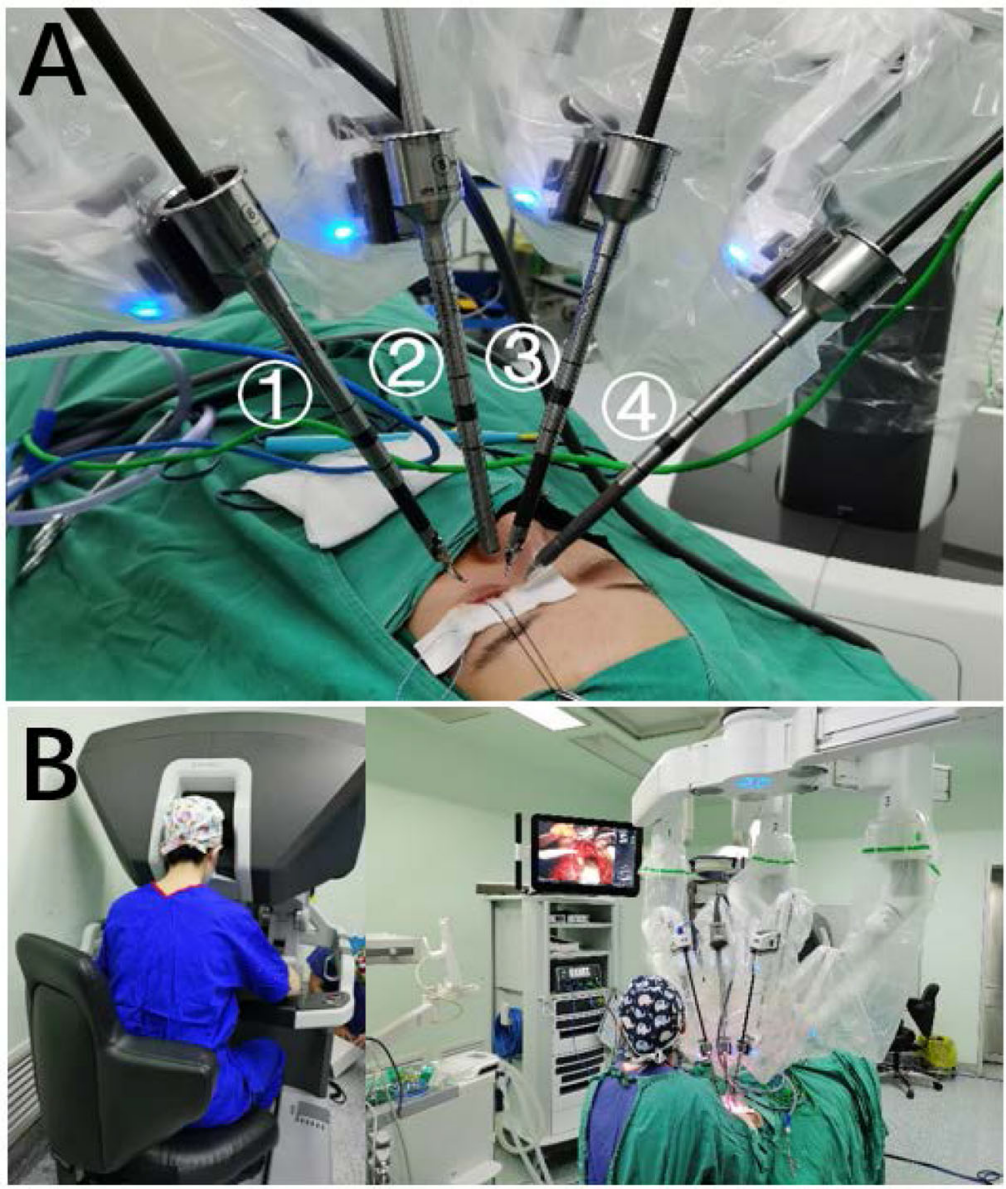
Work mode of da Vinci Xi. Robotic arm 1 was equipped with a curved bipolar dissector (da Vinci Xi 470344) and, respectively, 2: the 8-mm 0° endoscope (da Vinci Xi 470026), 3: Black Diamond micro-forceps (da Vinci Xi 470033), and 4: monopolar curved scissor (da Vinci Xi 470179). Arm 2 was approximately vertically placed above the eye. Arms 1 and 3 were placed in a bilateral symmetry, both with an angle of about 30° from arm 2. Arm 4 was on the far side, with an angle of about 60° from arm 2. The mean distance from the trocar to the eye was 10.15, 10.25, 10.25, and 10.4 cm for arms 1 to 4, respectively (**A**). The surgeon was seated in front of the robot console, away from the operating table, where one assistant was needed to cooperate with the robot (**B**).

With the patient under general anesthesia, we first docked the instrument cart to the appropriate position. The overall spatial arrangement of the operating room is shown in Figure 2. After disinfection of head and face exposure, the orbital fat in the infraorbital region was artificially exposed. After preparation of the surgical field, the robotic arms were equipped with an endoscope first to complete the targeting process and then the other three surgical tools, which are shown in Figure 3A. While the surgeon used the console to perform orbital surgery, the assistants were responsible for exposing the surgical field, suctioning blood, and monitoring intraoperatively (Fig. 3B).

**Figure 4.**
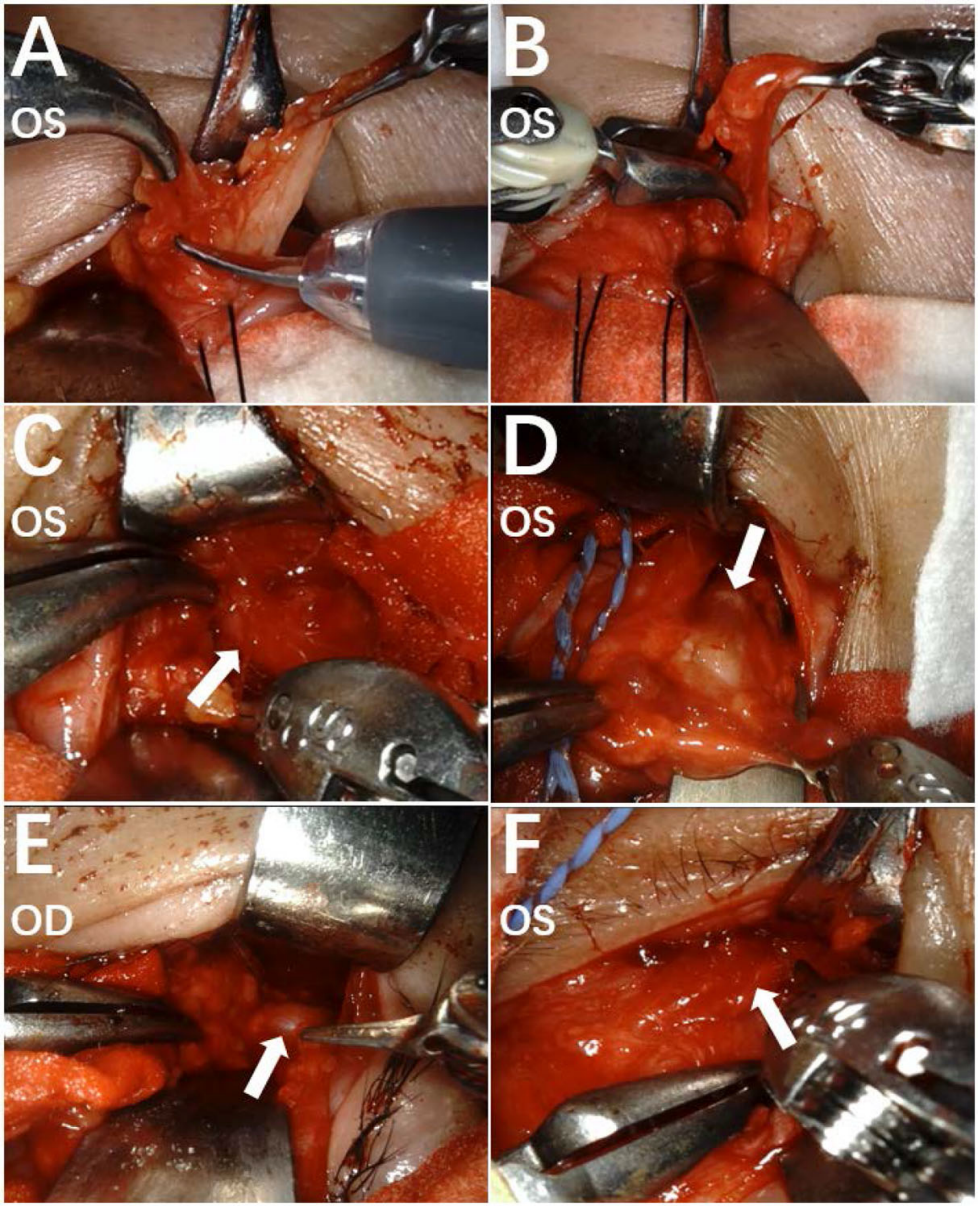
The intraoperative view of the surgical field by the endoscope. Orbital fat was excised by monopolar curved scissor (**A**), grasped by a curved bipolar dissector, and separated by Black Diamond micro-forceps (**B**) to accomplish full removal. The extraocular muscles were clearly identified and protected during the operation, including inferior rectus muscle (**C**, *white arrow*), medial rectus muscle (**D**, *white arrow*), lateral rectus muscle (**E**, *white arrow*), and inferior oblique muscle (**F**, *white arrow*).

When operating on superficial orbital fat, the curved bipolar dissector controlled by the left hand was used to grasp the fat, and Black Diamond micro-forceps (Worldwide Headquarters of INTUITIVE SURGICAL, 1020 Kifer Road, Sunnyvale, CA) controlled by the right hand were used for blunt separation so that the fat tissue could be pulled out. Then, the fat tissue was lifted using instruments in both hands and cut with monopolar curved scissors (Fig. 4A). When operating on deep orbital fat, malleable plates and neural pads were used to protect the eyeball and expose the surgical field. The fat from the medially and laterally inferior periocular part and within the eye muscle cone was removed in a “left-hand grasp and right-hand separation” way (Fig. 4B). The extraocular muscles were carefully separated and protected (Figs. 4C–F). The volume of fat removed was measured by a syringe, and the procedure was stopped when the volume removed reached the preoperative plan and good regression of exophthalmos was verified. Afterward, the defect was closed discontinuously with 6-0 absorbable thread. The pressure dressing was set with tobramycin dexamethasone ophthalmic ointment. Throughout the operation, gentle handling was ensured, and excessive pulling was avoided. All procedures were timed and videotaped (see Supplementary Video S1).

After the operation, methylprednisolone was given intravenously 2 days for anti-inflammatory purposes. Tobramycin and dexamethasone eye ointment was applied to the operated eye(s) daily after careful check and cleaning during hospitalization. After discharge, the operated eye(s) received levofloxacin and sodium hyaluronate eye drops daily. At 3 months postoperatively, patients were informed to recheck their situation at the outpatient clinic.

### Statistical Analyses

The exophthalmos, GO-QoL, and other continuous parameters were presented as mean ± standard deviation (SD). The normality of data distribution was tested using the Shapiro–Wilk test. Paired *t*-test was used to compare the exophthalmos and GO-QoL on the appearance arm before and after the surgery. Respectively, the Wilcoxon test was used to compare GO-QoL on the visual function arm before and after the surgery. All statistical tests were two-sided and performed at the level of significance of α = 0.05. Statistical analysis was performed with commercially available software (SPSS 25.0 [SPSS, Inc., Armonk, NY] and Prism 5 [GraphPad Software, Inc., La Jolla, CA, USA]).

## Results

All surgical procedures were successfully performed. The da Vinci Xi Surgical System can be used to complete the main procedures of orbital fat decompression surgery safely. Human assistance was needed to expose the surgical field, protect the eyeball, and clean up blood.

**Figure 5.**
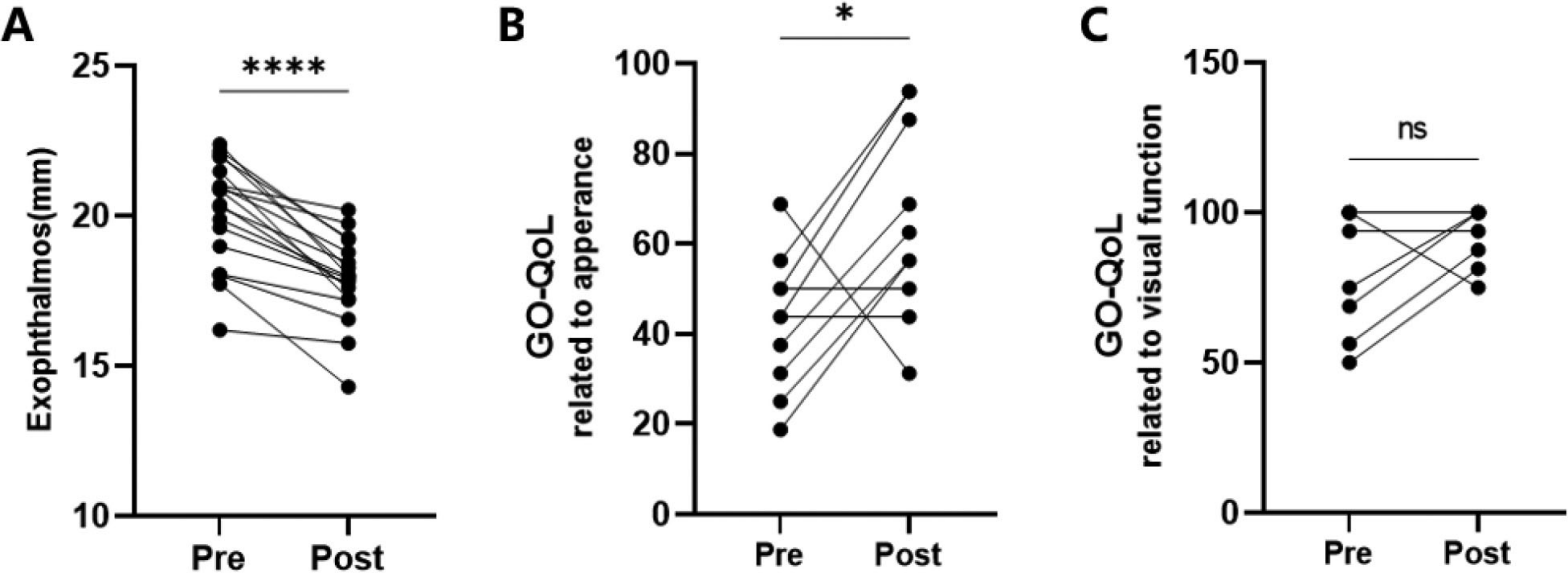
The paired self-comparison analyses of pre- and postoperation exophthalmos and GO-QoL scores. The exophthalmos (**A**) and GO-QoL on the appearance arm (**B**) were significantly improved. There was no significant increase in GO-QoL on the visual function arm (**C**).

The mean exophthalmos were significantly reduced from 20.2 ± 1.8 mm before surgery to 17.9 ± 1.4 mm postoperatively (*P*<0.0001, Fig. 5). The GO-QoL scores increased significantly from 42.50 ± 14.97 to 64.38 ± 21.46 on the appearance arm (*P* = 0.0270, Fig. 5). An increase from 84.38 ± 20.04 to 93.75 ± 9.32 in GO-QoL scores on the visual function arm was also found, but there was no statistical significance (*P* = 0.1875, Fig. 5). The mean volume of fat excised by the da Vinci robot was 3.6 ± 1.0 mL per eye. The mean total operative time was 124.3 ± 33.2 minutes. The mean time required for the docking of the robot and the installation of the robotic arms was 12.2 ± 6.3 minutes. The mean robotic operative time was 101.3 ± 35.6 minutes (56.3 minutes for each eye). The mean intraoperative blood loss was 17.8 ± 6.2 mL. Detailed information is shown in the Table.

**Figure 6.**
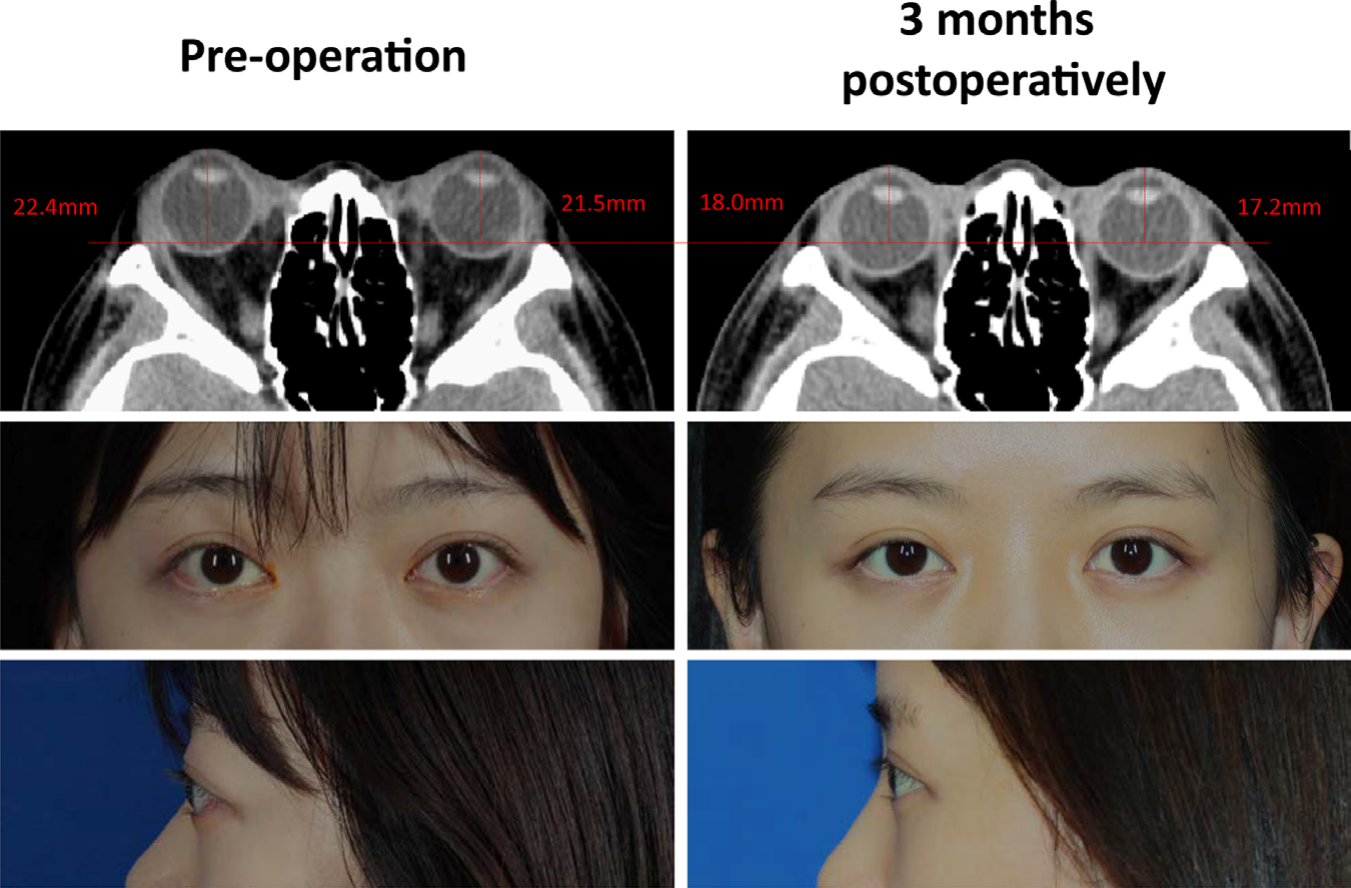
An overview of one patient with good curative effect. The orbital CT showed that the orbital fat tissue was reduced and both eyes were retracted into the orbit. The proptosis and eyelid retraction symptoms were both relieved after the surgery. A statement of consent to publish the results and images was gathered from the patient.

There were no intraoperative complications. No severe complications were found postoperatively. Three patients reported mild pain on the day after surgery and experienced self-recovery. Satisfying cosmetic outcomes were achieved (Fig. 6).

## Discussion

Ophthalmic surgery requires delicate and stable tissue manipulation, with special requirements for the experience and technique of the surgeon. Surgical robots have the potential to provide better visualization, stable manipulation, and increased precision, thus improving the safety and effect of surgery. The da Vinci surgical system is the most widely used commercially available surgical system today. Surgeons can control the surgical tools and equipped camera from a remote workstation using the robot, which can filter tremors and provide a three-dimensional view of the operative field with enhanced depth perception.^19^^–^^21^ Although it is not specifically designed for eye surgery, attempts at da Vinci robot-assisted ophthalmic surgery in experimental and clinical settings have shown feasibility in performing various surgical procedures.^15^^,^^22^^–^^26^ However, based on our review of the literature using EMBASE and MEDLINE databases, investigations concerning robotic orbital decompression surgery do not exist in the literature.

In our study, we successfully performed robot-assisted orbital fat decompression surgeries on 10 patients with GO. The mean volume of fat removed by Xi was 3.63 ± 1.00 mL. The mean regression of exophthalmos at 3 months postoperatively was 2.27 ± 1.21 mL. No severe complications were found. The mean amount of blood loss during the operation was 17.75 ± 6.22 mL. By using da Vinci surgical system, the surgical field was magnified 10 times to provide clear stereoscopic vision. Under endoscopy, tissues, such as fat, fascia, muscle, blood vessels, and conjunctiva, can be clearly distinguished, and depth can be perceived, which improves the safety of the operation. The surgery was easily performed with Black Diamond forceps, monopolar curved scissors, and micro-bipolar forceps, with no need of other specific microsurgical instruments. The extreme mobility of the distal articulation of the robotic arms holding the surgical instrument, motion scaling, and filtration of tremor can help with gentle operation and tissue damage reduction.

The mean total operative time was 124.30 ± 33.19 minutes. On average, it took 12.20 minutes to install the robot, the camera, and the instruments and 56.28 minutes to complete the robotic operation for each eye. Although it took longer for the surgery preparation, the actual operation time was not significantly different from that of traditional surgery according to our experience. Meanwhile, the surgeon cart of da Vinci provides the surgeons with a comfortable operating position, saving them from long-term heading down or shrugging in traditional orbital decompression surgery.^27^ Therefore, we think da Vinci can help relieve the surgeons’ fatigue. Moreover, the electrocoagulation function of curved bipolar dissector and monopolar curved scissor when grasping or cutting boosted the efficiency of surgery.

There was no significant increase in GO-QoL on the visual function arm postoperatively. We think this is because all enrolled patients had entered the inactive stage of GO and had a good basal visual function. Instead, the alterations in the appearance were what bothered them more. The GO-QoL on the appearance arm was significantly improved from 42.50 ± 14.97 to 64.38 ± 21.46, which was superior to the increase from 39.8 ± 26.2 to 56.3 ± 21.8 reported in traditional orbital fat decompression surgery.^28^

However, the disadvantage of da Vinci is the lack of applied force control and haptic feedback, limiting the accuracy of manipulation.^29^^–^^31^ In our previous study, we demonstrated the difficulty of performing orbital bony wall decompression in experimental settings using the skull model. The surgeon was unaware of the sense of breakthrough, which indicates detachment of the bone pieces and is vital for surgeons to stop further operations in a timely manner to avoid tissue damage and machine breakdown. Additionally, one assistant was needed for blood suction, protection of the eyeball, and intraoperative monitoring. The heavy workload of the assistant is also a disadvantage. The introduction of aspirators and retractors specially designed for orbital surgery to match the use of da Vinci may be helpful.

Clearly, small sample size and lack of control group in our study hold us from drawing a more precise conclusion. In the future, big-sample and controlled studies with higher evidence level are needed to validate what we, as an initial and exploratory study, had discovered about the advantage of da Vinci and justify the higher cost of the surgical system.^32^

## Conclusions

In this study, we explored the feasibility of performing da Vinci robot-assisted orbital fat decompression surgery. It was preliminarily discovered that da Vinci Xi provided the stability, dexterity, and good visualization necessary for orbital fat decompression surgery, and the exophthalmos and appearance of patients with GO were significantly improved. Further big-sample and controlled studies on the surgery are needed before it becomes a promising clinical option.

## Supplementary Material

Supplement 1

Supplement 2

Supplement 3

## Supplementary Video

Supplementary Video S1. An overview of da Vinci robot-assisted surgical procedures.
