# Supplementary material for: Robot-Assisted Orbital Fat Decompression Surgery: First in Human
Source: Transl Vis Sci Technol. 2022 May 10;11(5):8. doi: 10.1167/tvst.11.5.8 (PMC9100477; doi:10.1167/tvst.11.5.8)
Supplement: Supplement 1 [file tvst-11-5-8_s001.pdf]

Appendix 2

GO-QOL version UK1

The following questions deal specifically with your thyroid eye disease.
Please focus on the past week while answering these questions

During the past week, to what extent were you limited in carrying out the following activities, because of your thyroid eye disease?

Tick the box that matches your answer. The boxes correspond with the answers above them.
Please tick only one box for each question.

		Yes, seriously limited	Yes, a little limited	No not at all limited
1	Bicycling [never learned to ride a bike ☐]	☐	☐	☐
2	Driving [no driver's licence ☐]	☐	☐	☐
3	Moving around the house	☐	☐	☐
4	Walking outdoors	☐	☐	☐
5	Reading	☐	☐	☐
6	Watching TV	☐	☐	☐
7	Hobby or pastime, i.e.……………………..………..	☐	☐	☐

		Yes, severely hindered	Yes a little hindered	No not at all hindered
8	During the past week, did you feel hindered from something that you wanted to do because of your thyroid eye disease?	☐	☐	☐

The following questions deal with your thyroid eye disease in general

		Yes, very much so	Yes, a little	No,not at all
9	Do you feel that your appearane has changed because of your thyroid eye disease?	☐	☐	☐
10	Do you feel that you are stared at in the streets because of your thyroid eye disease?	☐	☐	☐
11	Do you feel that people react unpleasantly because of your thyroid eye disease?	☐	☐	☐
12	Do you feel that your thyroid eye diease has an influnce on your self-confidence?	☐	☐	☐
13	Do you feel socially isolated because of your thyroid eye disease?	☐	☐	☐
14	Do you feel that your thyroid eye disase has an influence on making friends?	☐	☐	☐
15	Do you feel that you appear less often on photos than before you had thyroid eye disease?	☐	☐	☐
16	Do you try to mask changes in appearance caused by your thyroid eye disease?	☐	☐	☐
