# Supplementary material for: Robot-Assisted Orbital Fat Decompression Surgery: First in Human
Source: Transl Vis Sci Technol. 2022 May 10;11(5):8. doi: 10.1167/tvst.11.5.8 (PMC9100477; doi:10.1167/tvst.11.5.8)

Pain Assessment Scale

Pain intensity（ Visual analogue scoring ）:

0———1———2———3———4———5———6———7———8———9———10

0 No pain　　1-3 Mild pain（Sleep is not affected）

4-6 Moderate pain（Sleep is affected）　　7-10 Serious pain（Sleep is severely affected）

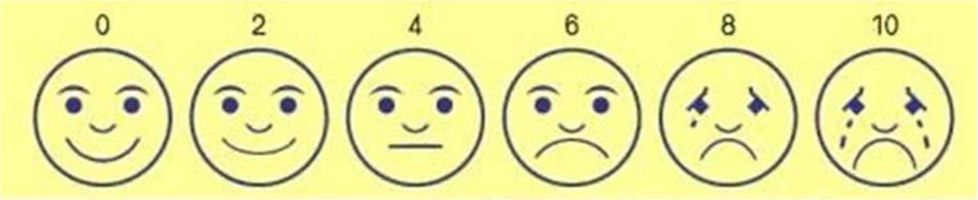

Supplement: Supplement 2 [file tvst-11-5-8_s002.pdf]
